# A quantitative measure of restricted and repetitive behaviors for early childhood

**DOI:** 10.1186/s11689-016-9161-x

**Published:** 2016-08-02

**Authors:** Jason J. Wolff, Brian A. Boyd, Jed T. Elison

**Affiliations:** 1 Department of Educational Psychology, University of Minnesota, Minneapolis, MN USA; 2 Department of Allied Health Sciences, University of North Carolina, Chapel Hill, NC USA; 3 Institute of Child Development, University of Minnesota, Minneapolis, MN USA

**Keywords:** Repetitive behavior, Motor stereotypy, Circumscribed interests, Ritualistic behavior, Self-injurious behavior, Measurement, Toddlers

## Abstract

**Background:**

Restricted and repetitive behaviors are characteristic phenotypic features of many neurodevelopmental, psychiatric, and neurological conditions. During early childhood, such behaviors are considered normative. More research is needed to delineate the dimensions of restricted and repetitive behavior across typical and atypical development during this period.

**Methods:**

We developed the 34-item parent-rated Repetitive Behavior Scale for Early Childhood (RBS-EC) to capture quantitative, dimensional features across a broad range of behaviors contributing to this domain. We evaluated its psychometric properties and factor structure in a community sample of 914 toddlers.

**Results:**

The RBS-EC showed excellent overall internal consistency (*α* = 0.90), strong test-retest reliability (ICC = 0.87 for topographies and 0.90 for frequency) and evidence of convergent and discriminative validity. Using a split-half approach to factor analysis, we identified that a three- or four-factor structure best fit the data and confirmatory factor analysis indicated acceptable fit for both models. The empirically derived four-factor model was consistent with our conceptual model and included repetitive motor, restricted interests and behavior, ritual and routine, and self-directed behavior.

**Conclusions:**

This initial study indicates that the RBS-EC is a reliable and valid instrument for characterizing quantitative, dimensional aspects of restricted and repetitive behaviors in young children.

**Electronic supplementary material:**

The online version of this article (doi:10.1186/s11689-016-9161-x) contains supplementary material, which is available to authorized users.

## Background

Restricted and repetitive behaviors (RRBs) denote a broad class of behavior characterized by repetition and invariance. This includes motor stereotypies, compulsive and ritualistic behaviors, repetitive self-injury, and inflexibility and rigidity, as well as circumscribed and intense interests or activities. Though a diagnostic feature of autism spectrum disorder, RRBs also contribute to the distinctive behavioral phenotypes of many etiologically defined as well as idiopathic neurodevelopmental disorders [1–3]. Restricted and repetitive behaviors are likewise associated with psychiatric and neurological conditions, such as stereotypic movement disorder, obsessive-compulsive and related disorders, Tourette’s syndrome, Parkinson’s disease, and anorexia nervosa [4–7]. The behavioral topographies constituting the domain of RRBs occur in patterns which may differentiate these diagnostic categories as well as cut across them [1–3, 8].

The presence of RRBs is not unique to pathological conditions. Such behaviors also occur over the entire course of normative human and nonhuman development [9–11]. This is most evident in early childhood, as typically developing children express a range of RRBs from infancy through school age. In human infants and toddlers, motor stereotypies are quite common and develop in progressive fashion, likely supporting the acquisition of increasingly sophisticated behavioral output [12–14]. Rigid and ritualistic patterns of behavior such as strict adherence to daily routines or insistence on sameness with regard to their environment and activities may be observed from toddlerhood through early school age [15, 16]. Other repetitive behavior topographies such as skin picking or finger/thumb sucking may continue into later childhood and have been associated with factors such as mood, arousal, and child maltreatment [17, 18]. While not conclusive, available evidence suggests that individual differences in early life restricted and repetitive behaviors may predict adaptive behavior, anxiety, temperament, and later emerging psychopathology [16, 19, 20].

A central challenge to better understand the relevance of early RRBs to clinical outcomes has been our inability to measure dimensionality in the manifestation of these behaviors, from typical to atypical, during a time of rapid development. Clinically oriented measures such as the Autism Diagnostic Interview-Revised [21] or Children’s Yale-Brown Obsessive Compulsive Scale [22], which include repetitive behavior items, are excellent for diagnostic purposes but are not designed to detect dimensional features of behavior that may be continuously distributed across individuals early in development. Automated [23–25] or direct-observation and video coding procedures [12, 14, 26, 27] are direct approaches to measuring RRBs which can yield highly dimensional data. However, these methods are typically costly and labor intensive and have generally been limited to measurement of motor stereotypies or self-injurious behaviors. Informant-based questionnaires are a viable alternative given that they are less costly to administer and more time efficient. Questionnaire measures of RRBs [15, 17, 28, 29], while more subjective and subject to bias, are also capable of capturing a wider range of behavioral topographies relative to direct measures.

The Repetitive Behavior Scale-Revised (RBS-R; [28]) is among the most widely implemented of such measures and has been used across a broad age range [30, 31], including early childhood [3, 13, 32, 33]. The RBS-R is comprised of 43 items, with each gauging presence or absence of behavior and its perceived severity. The measure groups items into six subscales, though several independent analyses have suggested alternative factor structures [31, 32, 34]. There are limitations to the RBS-R in early childhood samples, however, as it is ultimately a clinical severity measure which yields scores characterized by significant floor effects among typically developing children or children for whom a clinical condition may not be obvious or fully manifest [33].

In this study, we describe a newly developed parent- or-caregiver rated measure of restricted and repetitive behaviors specifically designed for use in early childhood. Our goal was to create a measure that was similar in structure to the RBS-R but tailored for use in early childhood. We further sought to maximize dimensional yield by constructing a measure that captures ordinal frequency information across the many behavioral topographies comprising the repetitive behavior domain. Here, we report initial psychometric and factor analyses of this new measure based on data collected from a community sample of 914 toddlers.

## Methods

### Measure development

Measure development was initiated and led by the first author in consultation with the second. Items were first derived from the RBS-R [28] and subsequently pooled with those from two versions of the Repetitive Behavior Questionnaire [29, 35] to check for construct overlap and to identify potential additional items. Nearly all items appearing on the Repetitive Behavior Questionnaire were deemed to have a counterpart item on the RBS-R with the exception of one item concerning unusual visual inspection of objects. Items were retained for revision based on the authors’ previous experience and if they were consistent with published literature reporting or describing such behaviors in typically developing children or children with a neurodevelopmental disorder [12, 14–17, 19, 29, 33, 36–38]. Items were excluded if their content was deemed age inappropriate, such as item 26 from the RBS-R regarding inflexibility related to travel and transportation. Some items reflecting more adult-oriented compulsive behavior from the RBS-R were also excluded given developmental relevance and evidence of significant floor effects among young children [33]. This included compulsivity items reflecting excessive cleaning, checking locks or doors, or counting. The revision process of items retained from the RBS-R included changes to terminology and/or item descriptions, such as the inclusion of the descriptors related to toys, “melt downs,” or other age appropriate objects and activities. For example, item 36 from the RBS-R, “Likes same CD, tape, record…” was revised as “Restricted use of media.” with reference to more contemporary media commonly used by children, such as mobile apps. New items were developed upon the basis of published work characterizing repetitive behaviors among infants, toddlers, and preschool/school aged children with and without a neurodevelopmental disorder [12, 14, 15, 33, 36]. This included items related to inflexible social interactions, unusual visual inspection, restricted movement or stillness, mouthing of objects, and more refined categories of motor stereotypies. These initial development steps culminated in a measure comprised of 41 items.

In order to better capture dimensionality among children for whom some degree of RRBs is expected, we avoided the use of clinical terminology or the framing of RRBs as problem behavior. All item descriptions were written to be consistent across the measure, developmentally appropriate for young children based on the authors’ expertise in repetitive behaviors and child development, and neutral with regard to behaviors as disruptive. Terms such as “stereotyped,” “compulsive,” “self-injurious,” and “sameness” were replaced with language deemed less clinical. In place of the four-level severity scale used by the RBS-R, a five-level rating scale was developed to gauge frequency of occurrence for each item over the previous month. This scale consisted of the following: 0-behavior does not occur, 1-behavior occurs about weekly or less, 2-behavior occurs several times a week, 3-behavior occurs about daily, and 4-behavior occurs many times a day. Each item contributes to two measures: topographies endorsed and frequency score. These measures may be summed into total or conceptually derived subscale scores. Initial subscales included repetitive motor, ritual and routine, restricted behavior, and self-directed behavior. Self-directed items reflect behaviors which may be considered self-injurious in some contexts but that in early childhood may not be associated with tissue damage or a behavioral disorder per se [17, 39].

Following item development by the study authors, content and face validity were assessed by obtaining outside feedback from experts in RRBs as well as overlapping areas/constructs including early child development, motor development, measurement, and autism spectrum disorder. Content experts were contacted by email and were asked to review the measure and respond to a set of open-ended survey questions. Feedback was also elicited from a group of parents (*n* = 8) who read an initial version of the measure, completed a brief set of open-ended questions, and discussed its content with the first author. Based on this external feedback, the measure was revised yielding the version presently under study—the Repetitive Behavior Scale for Early Childhood (RBS-EC). Approval for duplicate use of the name “Repetitive Behavior Scale” was obtained from the primary author of the RBS-R. Instructions accompanying the RBS-EC, as well as sample items, are provided in Additional file 1.

### Participants and procedures

Data collection occurred between May and October 2015. Parents of toddlers between 17 and 25 months of age, recruited from the Institute of Child Development (ICD) research participant registry, were invited to participate in a larger study about their child’s development. All parents of 17–25 months old during the study period were invited to participate, unless they had a toddler in the age range who was an active participant in an ongoing study conducted by ICD faculty or if their toddler had participated in a study in the previous 6 months. The ICD registry personnel acquire Minnesota birth records and invite parents to voluntarily select into the ICD Infant Participant Pool. The registry largely reflects the racial/ethnic proportions of the broader Minneapolis-St. Paul metropolitan area but under-represents the socio-economic diversity of this region. As part of the larger study, parents were invited to complete a form characterizing demographic and family characteristics, the Video-Referenced Rating of Reciprocal Social Behavior (vr-RSB) [40], the RBS-EC, and the MacArthur-Bates Communicative Development Inventories [41], in that order. All data collection occurred online [42], and it took parents ~45 min to complete the entire survey battery. Following the extant literature on survey research strategies [43], 2–3 days before receiving the online consent and questionnaires via email, parents were sent an introductory email describing the research. Parents were reimbursed with a $10 electronic gift card, and their name was entered into a drawing for a $50 electronic gift card (1 per 150 completed surveys) if they completed all of the questionnaires. Two follow-up emails were sent, 1- and then 2-weeks following the initial email, inviting parents to participate OR to complete forms if they had started but not finished the survey battery. All parents provided permission and informed consent to participate in the study. There were no exclusion criteria. Of the 2486 parents invited to contribute, 933 (38 %) completed the vr-RSB and RBS-EC questionnaires. As compared to the demographic characteristics of the children between 2 and 60 months in the ICD Infant Participant Pool, those who completed the surveys reported similar proportions of race/ethnicity classifications, but a higher household income (i.e., a lower proportion of responders reported household incomes between 25 and 75 K and a greater proportion of responders reported household income more than 200 K). Characteristics of the sample are given in Table 1. Nineteen participants were excluded for providing responses that suggested invalid data (e.g., time to complete the entire in survey was less than 1 min). Study procedures were approved by the University of Minnesota Institutional Review Board (#1501S61261) and informed written consent was obtained from each participant.

**Table 1:** Descriptive and demographic data for study sample

Variable	Number	Percent	Mean	(SD)
Age in months	914		19.7	(2.4)
Gestational age (days)	783^a^		275.2	(14.2)
Birth weight (grams)	895^a^		3493	(540)
Sex				
Females	442	48		
Males	472	52		
Race/ethnicity				
White	814	89		
Non-white or mixed	100	11		
Parent’s education				
Some college or less	113	12		
College degree	429	47		
Graduate degree	372	41		

^a^Gestational age and birth weight data not available for all cases

### Parent-report questionnaires

The *Video-Referenced Rating of Reciprocal Social Behavior*, version 2.3 [40] represents a downward extension of the Social Responsiveness Scale, second edition (SRS-2) [44]. The first 13 items (out of a total of 48 items) are scored in reference to a 3-min video of a typically developing 19 month-old child demonstrating a variety of reciprocal social behaviors (e.g., expressing feelings through changes in facial expression, cooperating with adult’s request, and performing showing and requesting behaviors). Thirty-five additional questions reflect quantitative aspects of social behavior and two final questions ask the parent to report specifically about the number of words produced by the child and to provide an example of a complex/sophisticated sentence produced by their child. The instrument takes ~15–20 min to complete. An initial study documented appropriate test-retest reliability, modest convergent validity with a measure of social communicative development and high concordance among monozygotic twins as compared to dizygotic twins [40]. As previously reported, a total summary score and a summary score of the video-referenced items only can be derived. The vr-RSB items can also be conceptually grouped into social and repetitive domains (correspondence between the JTE and N. Marrus), and these domain scores, along with video-referenced reciprocal social behavior scores, were derived to examine convergent and divergent validity with the RBS-EC. As the focus of this analysis is on the initial description of the RBS-EC, a full analysis of the vr-RSB data will be described in a subsequent publication.

The Repetitive Behavior Scale for Early Childhood has been described above.

### Analysis

Mann-Whitney *U* tests were used to examine sex differences in RBS-EC total and subscale scores. Test-retest reliability was assessed by intraclass correlations of data from a subset (*n* = 46) of the total sample. Internal consistency of RBS-EC composite and subscale measures was assessed by Chronbach’s alpha. Convergent and divergent validity were assessed using correlations between overall scores from the RBS-EC and separate measures of social and repetitive behavior from the vr-RSB. Because data did not meet the primary assumptions for parametric analysis, Spearman rank-order correlations were used.

To further assess construct validity, we employed a two-stage factor analytic strategy, starting with exploratory and proceeding to confirmatory factor analysis using a split-half approach. Exploratory factor analysis (EFA) was conducted on the first split-half to characterize the item-level factor structure of the RBS-EC. These analyses used a maximum likelihood extraction method with oblique (promax) rotation. To determine the number of factors to extract, scree plots were generated and examined. Next, we used confirmatory factor analysis to test models derived from the EFA. A cutoff of 0.35 was used to determine items retained within models from the EFA. We also tested the conceptually derived model which formulated the original subscales of the RBS-EC. Model fit statistics include the comparative fit index (CFI), root mean square error of approximation (RMSEA), and standardized root mean square residual (SRMR). Models with adequate-to-good fit are indicated by CFI close to 0.95 (or >0.90), RMSEA <0.60, and SRMR <0.80 [45, 46]. Analyses were performed using IBM SPSS, version 23.

## Results

Table 1 provides descriptive and demographic data for the study sample. Of the total study sample (*n* = 914), 442 were female (48 %). The average age of participants was 19.7 months (*SD* = 2.4) with a range of 17 to 27 months. Gestational age data were available for 86 % (*n* = 783) of our sample, and approximately 9 % (*n* = 70) of these children had gestational ages of ≤36 weeks.

The average RBS-EC composite topography score for the sample was 12 (*SD* = 6.7), and the average composite frequency score was 24.6 (16.7). Males and females differed significantly in composite frequency score (*U* = 95,833, *Z* = 2.1, *p* = 0.03) but not composite topographies (*U* = 98,436, *Z* = 1.5, *p* = 0.14).

Among subscales, there were sex differences in restricted behavior frequency (*U* = 92,697, *Z* = 2.9, *p* = 0.003) and topographies (*U* = 93,916, *Z* = 2.6, *p* = 0.008), with males having higher scores. There were no sex differences in scores for the other three subscales (all *p* > 0.12). Complete RBS-EC total and subscale scores are presented in Table 2. Graphed response distributions for subscale topographies endorsed and frequency scores are presented in Additional file 2: Figures S1 and Additional file 3: Figure S2.

**Table 2:** Repetitive Behavior Scale for Early Childhood composite and subscale scores

	Scoring scale	Total sample (*n* = 914)		Females (*n* = 442)		Males (*n* = 472)	
RBS-EC scale	Min/max	Mean	SD	Mean	SD	Mean	SD
Composite topographies	0/34	12.0	6.7	11.6	6.8	12.3	6.6
Repetitive motor topographies	0/9	5.8	3.0	5.7	3.1	5.9	3.0
Ritual and routine topographies	0/10	2.0	2.0	2.0	2.1	2.0	1.9
Restricted topographies	0/8	2.6	2.2	2.4^a^	2.2	2.7^a^	2.2
Self-directed topographies	0/7	1.6	17	1.6	1.8	1.6	1.6
Composite frequency	0/136	24.6	16.7	23.3^a^	16.1	25.8^a^	17.1
Repetitive motor frequency	0/36	15.2	11.2	14.6	11.3	15.7	11.2
Ritual and routine frequency	0/40	2.9	3.5	2.7	3.1	3.1	3.8
Restricted frequency	0/32	4.3	4.4	3.8^a^	4.1	4.7^a^	4.7
Self-directed frequency	0/28	2.2	2.7	2.1	2.7	2.2	2.7

^a^Sex difference statistically significant at *p* < 0.05

### Reliability

Test-retest data were available for 5 % (*n* = 46) of the total sample. Retesting was completed within 3 weeks of the initial administration, with the median retesting taking place at 7 days out (range 1 to 19 days). Intraclass correlation coefficients between scoring administrations were 0.87 for total score (topographies endorsed) and 0.90 for total frequency score. With regard to internal consistency, Chronbach’s alpha for the RBS-EC as a whole was 0.90. Among the four conceptually derived subscales, Chronbach’s apha values were all within the acceptable range with 0.93 for repetitive motor (9 items), 0.75 for ritual and routine (10 items), 0.77 for restricted behavior (8 items), and 0.70 for self-directed (6 items).

### Construct validity

In terms of convergent validity, repetitive behaviors measured by the vr-RSB significantly correlated with total composite score (*r*_*s*_ = 0.43, *p* < 0.001) and total frequency score (*r*_s_ = 0.39, *p* < 0.001) on the RBS-EC. For discriminant validity, summary scores from video-referenced items on the vr-RSB did not correlate with total composite or total frequency scores from the RBS-EC (*r*_s_ < 0.02, *p* > 0.65). However, both the total composite and total frequency scores from the RBS-EC did correlate weakly but statistically significantly with social-communication summary scores from the vr-RSB, *r*_s_ = 0.17, *p* < 0.001 (note that higher scores on the vr-RSB reflect less sophisticated social-communication).

### Factor structure

There were no differences between the split-half samples in terms of age (*t*(912) = 0.8, *p* = 0.4), sex (Fisher’s exact test; *p* = 0.95), or repetitive behavior composite score (*t*(912) = 0.7, *p* = 0.5). For the initial EFA sample (*n* = 457), the KMO index indicated excellent overall sampling adequacy (KMO = 0.91). Visual inspection of scree plots suggested that either a three- or four-factor solution were most appropriate for the data. Rotated factor solutions for these models are presented in Tables 3 and 4.

**Table 3:** Rotated pattern matrix for four-factor solution from exploratory factor analysis

Item heading	Repetitive motor	Ritual and routine	Restricted	Self-directed
Torso	*0.93*	0.04	−0.01	0.01
Head	*0.88*	−0.02	−0.01	−0.03
Legs	*0.85*	−0.06	0.03	−0.05
Arms, hand, or fingers	*0.84*	0.07	0.03	−0.04
Arms/hand/fingers on surfaces	*0.81*	0.00	0.02	0.03
Locomotion	*0.79*	0.00	0.00	−0.02
Object use	*0.72*	−0.11	0.05	0.03
Vocalizations	*0.70*	0.01	0.09	0.06
Mouthing objects	*0.50*	0.04	−0.03	0.15
Play	−0.02	*0.78*	−0.02	0.00
Daily routine	−0.04	*0.73*	−0.04	0.06
Change in others	0.01	*0.66*	−0.02	0.03
Mealtime	0.06	*0.61*	−0.01	0.03
New places	−0.09	*0.58*	0.10	0.05
Bedtime	0.05	*0.54*	0.06	−0.06
Social interactions	−0.05	*0.53*	0.10	0.09
Interruptions	0.02	*0.40*	0.13	0.13
Visual inspection	−0.03	−0.08	*0.76*	0.09
Fascination with movement	0.02	−0.13	*0.71*	0.18
Interest in parts of objects	−0.04	−0.08	*0.67*	0.16
Limited and intense interest	0.04	0.05	*0.48*	0.04
Stillness	−0.01	0.10	*0.47*	−0.14
Sensory interests	0.09	0.13	*0.45*	−0.01
Media use	−0.01	0.22	*0.40*	−0.08
Object attachment	−0.02	0.14	*0.40*	−0.02
Placement of objects	0.04	0.32	*0.35*	−0.19
Arranging toys	0.13	0.08	*0.32*	−0.18
Hits self with object	0.01	0.05	−0.03	*0.73*
Hits self with body part	0.09	0.08	−0.20	*0.72*
Hits self against surface	0.01	0.01	0.02	*0.63*
Pulls own hair	−0.02	0.14	0.04	*0.32*
Scratches, pinches, or pokes self	0.01	−0.09	0.20	*0.32*
Bites self	−0.05	0.02	0.10	*0.19*
Skin picking	0.00	0.11	0.07	*0.14*

Italics signify items belonging to a common factor

**Table 4:** Rotated pattern matrix for three-factor solution from exploratory factor analysis

Item heading	Repetitive motor	Ritual and routine	Restricted
Torso	*0.94*	0.04	−0.11
Head	*0.89*	−0.03	−0.03
Legs	*0.84*	−0.08	0.00
Arms, hands, or fingers	*0.84*	0.06	0.00
Arms/hands/fingers on surfaces	*0.81*	0.00	0.03
Locomotion	*0.78*	0.00	−0.02
Object use	*0.73*	−0.11	0.06
Vocalizations	*0.71*	0.02	0.11
Mouthing objects	*0.52*	0.06	0.03
Play	−0.03	*0.80*	−0.06
Daily routine	−0.04	*0.77*	−0.06
Change in others	0.01	*0.68*	−0.04
Mealtime	0.06	*0.63*	−0.03
New places	−0.09	*0.60*	0.09
Social interactions	−0.04	*0.56*	0.12
Bedtime	0.04	*0.54*	0.01
Interruptions	0.03	*0.43*	0.16
Placement of objects	0.00	*0.29*	0.25
Hits self with body part	0.21	*0.22*	0.11
Pulls own hair	0.03	*0.21*	0.18
Skin picking	0.01	*0.17*	0.12
Visual inspection	−0.04	−0.07	*0.82*
Fascination with movement	0.03	−0.11	*0.80*
Interest in parts of objects	−0.03	−0.06	*0.74*
Limited and intense interests	0.04	0.05	*0.49*
Sensory interests	0.08	0.13	*0.44*
Stillness	−0.05	0.07	*0.40*
Object attachment	−0.04	0.13	*0.38*
Media use	−0.03	0.20	*0.35*
Scratches, pinches, or pokes self	0.05	−0.03	*0.34*
Hits self against surface	0.11	0.14	*0.28*
Hits self with object	0.13	0.20	*0.27*
Arranging toys/objects	0.10	0.04	*0.23*
Bites self	−0.02	0.06	*0.19*

Italics signify items belonging to a common factor

The four-factor model was similar in composition to the conceptually derived model, with the exception of two items originally designated as “ritual and routine” loading on the “restricted behavior” factor (object arrangement, placement of objects). The three-factor model was similar in composition to the four-factor model but with items originally designated as “self-directed” loading with either “ritual and routine” or “restricted behavior.” To assess the fit of the three- and four-factor models, two- and five-factor models were also generated. The two-factor model was characterized by numerous cross-loading items and is not presented here. The five-factor model resulted in a pattern matrix resembling that for the four-factor model but with a fifth factor consisting of a single item that previously loaded with repetitive motor (repetitive use of toys and other objects).

Four models were subsequently tested through CFA on the second split-half sample (*n* = 457). Three models were derived from the EFA (four-, three-, and two-factor models), while the fourth was based on the original conceptually derived structure of the RBS-EC. The five-factor model tested in the EFA phase was not examined through CFA given that one of its factors comprised a single item. Goodness-of-fit statistics for these four-factor models are presented in Table 5.

**Table 5:** Confirmatory factor analysis model fit parameters for RBS-EC

Factors	*χ* ^2^	*df*	*p*	CFI	RMSEA	SRMR
4^a^	1181.2	489	<0.001	0.88	0.056	0.057
4	795.4	344	<0.001	0.91	0.054	0.048
3	674.3	272	<0.001	0.92	0.057	0.048
2	976.6	298	<0.001	0.86	0.071	0.070

*CFI* comparative fit index, *RMSEA* root mean square error of approximation, *SRMR* standardized root mean square residual

^a^Conceptually derived four-factor model with all items

Overall, test indices indicated acceptable fit for the EFA-derived four-factor and three-factor models. The conceptually derived four-factor model, which included all original items, was acceptable in terms of RMSEA and SRMR statistics but just below the recommended range for the CFI. Fit statistics indicated that the two-factor model should be rejected.

## Discussion

**Fig. 1:**
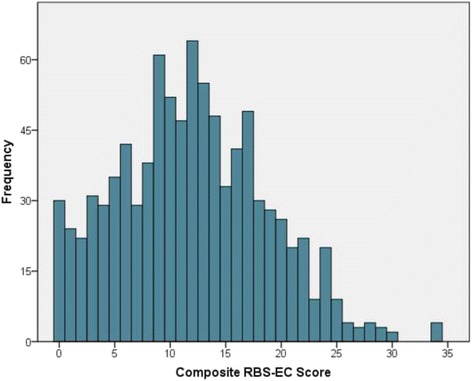
Response distribution for total topographies endorsed

We developed a parent-report measure of restricted and repetitive behaviors (RRBs), specifically designed for use in early childhood, based on the general structure of the widely used Repetitive Behavior Scale-Revised [28]. Our intention in so doing was to create a developmentally appropriate behavioral measure capable of capturing quasi-dimensional information across a broad range RRBs. Once developed and assessed for content validity, we tested our 34-item measure (RBS-EC; Repetitive Behavior Scale for Early Childhood) in a community sample of 914 toddlers. The results suggest that the RBS-EC has good psychometric properties including strong overall internal consistency, adequate-to-strong internal consistency among conceptually derived subscales, and strong test-retest reliability. As with previous studies of RRBs among young children [15, 16, 29, 47], we observed a wide range of response patterns which, as measured in total by the RBS-EC, suggest a high degree of inter-individual variability (Fig. 1).

Factorial validity was assessed by a split-half approach balancing exploratory and confirmatory factor analysis. We identified that a three- or four-factor model best fit the data, with the latter aligning well with the original conceptual organization of the measure. It would appear that the RBS-EC may be used with or without the subscale measuring self-directed behaviors (four or three factors, respectively). Inclusion of this subscale may be of particular relavance to children with or at-risk for a neurodevelopmental disorder given that self-injurious behavior is both more common and a likely target of intervention/prevention among this population [2, 3, 13]. When considered along with initial content validity checks, the results of the factor analyses suggest that test scores from the RBS-EC reflect the construct of restricted and repetitive behavior. Construct validity of the RBS-EC was further evidenced by preliminary tests of convergent and discriminant validity. Scores from the measure significantly correlated with repetitive behavior scores derived from the vr-RSB [40] but showed little overlap with its video-referenced social behavior subscale. We did observe, however, a modest but statistically significant correlation between the RBS-EC and the social-communication summary score from the vr-RSB. The effect size of this relationship was relatively weak, accounting for less than 5 % of shared variance. However, while potentially an artifact related to sample size, there is some evidence that RRBs and social-communication skills may be inversely associated during toddlerhood [27, 33], and conceptually, there is little reason to assume a purely orthogonal relationship between these constructs.

Despite their ubiquity in early childhood, there is evidence that restricted and repetitive behaviors may differentiate children with and without developmental concerns such as autism during toddlerhood [26, 27, 33, 36, 48] and as early as the first year of life [49, 50]. What is not clear is whether RRBs associated with early atypical development belong to the same class of behavior as those associated with typical development. Stated differently, are repetitive behaviors associated with atypical early development the extreme end of a continuum of behavioral output characteristic of all children, or do they qualitatively differ with regard to underlying function and mechanism? While we cannot speak directly to these questions, data from the present study provides some support to the position that repetitive behaviors in early childhood represent a dimensional behavioral feature given that we observed a continuum of RRBs in our sample of toddlers. This point is illustrated by the distribution of responses shown in Fig. 1. For those children with or at risk for neurodevelopmental disorders, the expected pattern of RRBs may be pronounced, follow an altered trajectory, and fail to resolve as more adaptive behaviors come online [13, 20]. We posit that these children and others who are atypically developing will fall disproportionately into the right tail of distribution illustrated in Fig. 1. Follow-up work will be required to fully address this issue.

The RBS-EC was developed for use in early childhood, acknowledging the behavioral variability that occurs between infancy and school age. While some aspects of this scale may be appropriate for use in a child 1 year old or younger—the repetitive motor subscale, for example, might be applied to children as young as 1–2 months [12, 14]—others would clearly not be applicable to this age group. We expect items such as “restricted use of media” to be quite relevant to the behavioral repertoire of preschool and elementary school aged children but substantially less so for infants. As the present results pertain only to toddlers, the upper- and lower-age limits of the RBS-EC are unknown. We designed the measure considering a provisional upper-age limit proximal to early school age. This was based in part on previous reports suggesting that RRBs among most children wane after age 5 and reach negligible levels by early adolescence [15, 17, 51, 52]. However, as there is very little published literature pertaining to normative patterns of RRBs in school aged children, it is difficult to estimate the suitability of the RBS-EC for this age group. It will therefore be necessary to directly assess the validity and psychometric properties of the RBS-EC outside of toddlerhood to establish developmental variation in factor structure as well as upper and lower-age boundaries.

Some conceptual models of RRBs have grouped repetitive motor behaviors with self-directed (or self-injurious) behaviors, describing these as “lower order” or “sensory-motor” given shared qualities related to topography [53]. There is also a long-standing hypothesis that motor stereotypies may function as precursors of self-injury among children with or at-risk for a neurodevelopmental disorder [54, 55], and there is some empirical support to this effect [56]. In this study, however, we did not identify any circumstance under which these subtypes of repetitive behavior loaded together. Indeed, when items were constrained to a two-factor model, the majority of self-directed items loaded with ritualistic and routine behaviors. Our results are consistent with other studies finding that self-injurious behaviors occur in typically developing toddlers absent elevated stereotypy [57] and that these subtypes of RRB are a distinct class of behavior from motor stereotypy in children with autism [31, 32]. It is possible that the construct of proto-self-injurious behavior has been over-extended to include *any* motor stereotypy, when a more narrow definition related to body-directed, pre-injurious topographies is warranted [39].

The present work was based on a community sample of toddlers. It is likely that these children are characterized by a continuum of cognitive and adaptive functioning and not just so-called typical development. For example, our sample includes a range of birth weights and gestational ages that reflect the US population. However, we cannot be certain of the cognitive or adaptive behavioral characteristics of our sample as these measures were not collected. We are therefore unable to speak to the relationship of RRBs to such variables. Follow-up work should collect additional cognitive and behavioral data as well as enrich for target populations who have been identified as having a neurodevelopmental disorder. Doing so would help clarify where individuals characterized by atypical development fall on the continuum of RRBs measured by the RBS-EC and contribute to assessment of possible clinical utility and criterion-related validity. This is an essential next step given that the present work cannot directly speak to the performance of the RBS-EC among children with neurodevelopmental disorders. Future work might also further assess construct validity by comparing the RBS-EC with similar measures such as the RBS-R or RBQ-2 [28, 29] and clarify upper- and lower-age limits among children who are typically developing as well as children with neurodevelopmental disorders.

## Conclusions

In most circumstances and for the majority of children, some degree of restricted and repetitive behavior is both adaptive and expected. There appears, as indicated by this and similar studies of young children, a continuum of such behavior in the general population. What is not yet known are the development implications of individual differences in these behaviors early in life. It has become increasingly clear that early deviations or excesses in RRBs may indicate risk for disorders of neurodevelopment. Improved characterization of patterns and trajectories of restricted and repetitive behavior during early childhood may enhance efforts to identify at-risk children and delineate clinically meaningful subgroups based on shared and specific phenotypes.

## Additional files

Additional file 1: Instructions and sample items from the RBS-EC. (DOCX 25 kb) Additional file 2: Figure S1. Response distributions for RBS-EC subscale topographies endorsed. (TIF 88 kb) Additional file 3: Figure S2. Response distributions for RBS-EC subscale frequency scores. (TIF 109 kb)

## Abbreviations

ASD: autism spectrum disorder
RBS-EC: Repetitive Behavior Scale for Early Childhood
RBS-R: Repetitive Behavior Scale, Revised
RRB: restricted and repetitive behavior
vrRSB: Video-Referenced Rating of Reciprocal Social Behavior
