# Supplementary material for: A quantitative measure of restricted and repetitive behaviors for early childhood
Source: J Neurodev Disord. 2016 Aug 2;8:27. doi: 10.1186/s11689-016-9161-x (PMC4970296; doi:10.1186/s11689-016-9161-x)
Supplement: Additional file 1: — Instructions and sample items from the RBS-EC. (DOCX 25 kb) [file 11689_2016_9161_MOESM1_ESM.docx]

**Additional File 1: Instructions and sample items from the RBS-EC**

**Instructions provided to parents/caregivers:**

This is a measure of repetitive behaviors for use in children from infancy through early school-age. Repetitive behaviors range from simple motor movements to complex patterns of interests and routines. Many of these behaviors are very common in children and occur as part of healthy development.

**INSTRUCTIONS:** Please rate your child’s behavior for each of the 34 items listed by circling the score that best describes how often the behavior occurs. Be sure to read and score all items. Make your ratings based on your child’s behavior over the past month. Use the definitions in the box given below to score each item:

**0 – behavior does not occur**

**1 – behavior occurs about weekly or less**

**2 – behavior occurs several times a week**

**3 – behavior occurs about daily**

**4 – behavior occurs many times a day**

If an item is “not applicable” because your child cannot yet perform a particular behavior (for instance, he or she is not yet grasping objects), the item should be scored as “0” (behavior does not occur). Please note that many items may not apply to infants given their limited range of behavior.

**Example items^1^ measuring Repetitive Motor:**

| ARMS/HANDS/FINGERS ON SURFACES (slaps, taps, or drums against objects, walls, floors, tables, or other furniture | 0 | 1 | 2 | 3 | 4 |
| --- | --- | --- | --- | --- | --- |
| OBJECT USAGE (bangs, spins, twirls, shakes, drops or rolls toys or other objects) | 0 | 1 | 2 | 3 | 4 |
| MOUTHING OBJECTS (mouths, bites, licks, or sucks objects – *do not count bottles, pacifiers, cups or utensils*) | 0 | 1 | 2 | 3 | 4 |

**Example items measuring Ritual and Routine:**

| SLEEPING/BEDTIME (refuses to sleep in new places; insists that room or bed is “just so” at bedtime; insists that specific activities precede bedtime) | 0 | 1 | 2 | 3 | 4 |
| --- | --- | --- | --- | --- | --- |
| PLAY (follows a strict play routine; insists that others play in a specific way; upset if play routine is altered) | 0 | 1 | 2 | 3 | 4 |
| SOCIAL INTERACTION (insists that others respond in a specific way; follows a set verbal script or routine regardless of context or social behavior of other children or adults) | 0 | 1 | 2 | 3 | 4 |

**Example items measuring Restricted Interests and Behavior:**

| LIMITED & INTENSE INTERESTS (narrow preoccupation with one subject or activity, e.g., trains, dinosaurs, collecting items, e.g. rocks, sticks, strings; plays only with specific toys) | 0 | 1 | 2 | 3 | 4 |
| --- | --- | --- | --- | --- | --- |
| RESTRICTED USE OF MEDIA (strongly insists on same music, book, app, program, movie or part of program/movie etc.; firmly refuses new books/apps/movies etc.) | 0 | 1 | 2 | 3 | 4 |
| SENSORY INTERESTS (seeks specific tactile, auditory, or visual sensations, e.g. smells or rubs specific objects; intense fascination with specific sounds, lights, or textures) | 0 | 1 | 2 | 3 | 4 |

**Example items measuring Self-Directed Behavior:**

| HITS SELF AGAINST SURFACE (hits or bangs head or other body part on furniture, walls, floors, or other surfaces) | 0 | 1 | 2 | 3 | 4 |
| --- | --- | --- | --- | --- | --- |
| BITES SELF (bites hand, fingers, arm, lips or tongue) | 0 | 1 | 2 | 3 | 4 |
| RUBS, SCRATCHES, POKES OR PINCHES SELF (*do not count itching of bug bites, eczema or other skin irritation*) | 0 | 1 | 2 | 3 | 4 |
