# Supplementary figures and images for: A quantitative measure of restricted and repetitive behaviors for early childhood
Source: J Neurodev Disord. 2016 Aug 2;8:27. doi: 10.1186/s11689-016-9161-x (PMC4970296; doi:10.1186/s11689-016-9161-x)

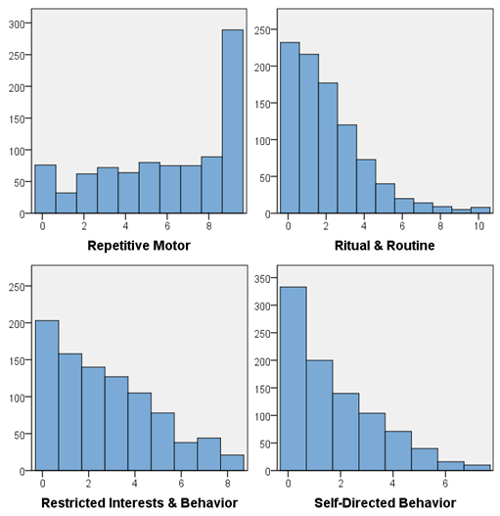

Supplement: Additional file 2: Figure S1. — Response distributions for RBS-EC subscale topographies endorsed. (TIF 88 kb) [file 11689_2016_9161_MOESM2_ESM.tif]

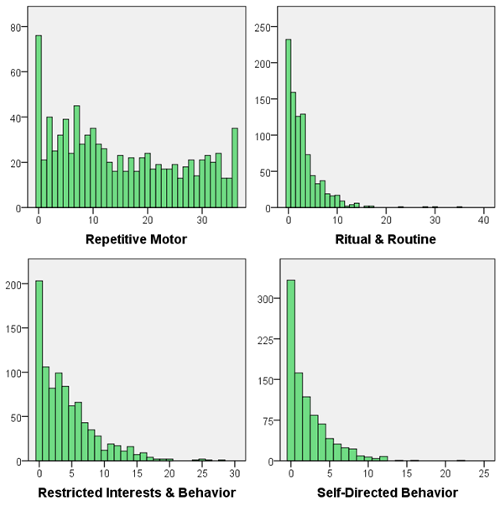

Supplement: Additional file 3: Figure S2. — Response distributions for RBS-EC subscale frequency scores. (TIF 109 kb) [file 11689_2016_9161_MOESM3_ESM.tif]
